# On-Scene Time-Dependent Probabilities of Return of Spontaneous Circulation and Favorable Neurological Outcome in Cases of Out-of-Hospital Cardiac Arrest with an Initial Shockable Rhythm: A Retrospective Study Using the National Utstein Data in Japan

**DOI:** 10.3390/jcm15218484

**Published:** 2026-10-31

**Authors:** Keita Miyazaki, Arisa Kinoshita, Koji Yamamoto, Keisuke Tsuruta, Hideki Asai, Yasuyuki Kawai, Keigo Saeki, Hidetada Fukushima

**Affiliations:** 1 Department of Emergency and Critical Care Medicine, Nara Medical University, 840 Shijo-cho, Kashihara 634-8521, Nara, Japan; 2 Department of Epidemiology, Nara Medical University, 840 Shijo-cho, Kashihara 634-8521, Nara, Japan

**Keywords:** out-of-hospital cardiac arrest, initial shockable rhythm, on-scene resuscitation, return of spontaneous circulation, neurological outcome

## Abstract

**Background/Objectives:** In out-of-hospital cardiac arrest (OHCA) with an initial shockable rhythm, the likelihood of achieving on-scene return of spontaneous circulation (ROSC) and favorable neurological outcomes changes with elapsed resuscitation time. We aimed to describe these time-dependent patterns under existing Japanese emergency medical services (EMS) practice. **Methods:** We conducted a nationwide retrospective descriptive cohort study using linked Japanese Utstein and emergency transport data from 2017–2022. Adults with cardiogenic OHCA and an initial shockable rhythm were included. For each time point after EMS contact, we calculated the observed probability of subsequent on-scene ROSC and, among patients achieving on-scene ROSC, the probability of a favorable neurological outcome (CPC 1–2 at 1 month) according to ROSC timing. **Results:** Among 22,383 eligible patients, 5095 achieved on-scene ROSC and 3182 had favorable neurological outcomes. The probability of subsequent on-scene ROSC was 6.97% (1295/18,583; 95% CI, 6.61–7.34%) at 10 min and 0.50% (87/17,375; 95% CI, 0.41–0.62%) at 20 min. At 10 min, favorable neurological outcomes occurred in 50.0% of patients aged <75 years and 20.5% of those aged ≥75 years who achieved on-scene ROSC at or after that time point. A sensitivity analysis restricted to 3128 patients receiving first EMS defibrillation within 1 min showed broadly similar temporal patterns. **Conclusions:** Under existing Japanese EMS practice, subsequent on-scene ROSC and favorable neurological outcomes became less frequent with later ROSC timing. Younger patients showed more favorable neurological outcomes at later ROSC times, but these findings do not establish age-based criteria for prolonged resuscitation or transport decisions.

## 1. Introduction

Out-of-hospital cardiac arrest (OHCA) is a major public health issue. The percentages of associated survival with favorable neurological outcomes remain at 3.5% and 8.1% in Japan and the US, respectively, despite the various initiatives undertaken [1,2]. Furthermore, the survival rate for OHCA with an initial shockable rhythm is 20–30% [1,2], and improving the outcomes in this context is an urgent requirement. Public access defibrillation has been reported to be effective in such cases of OHCA; however, its frequency remains <20% [2,3], and defibrillation by emergency medical services (EMS) accounts for most prehospital defibrillations. Regarding cases in which EMS performs on-scene resuscitation, a longer on-scene time is associated with a poor prognosis [4,5,6]. Several studies have examined intra-arrest transport versus continued on-scene resuscitation [7,8,9]. However, the temporal pattern of ROSC observed during on-scene resuscitation represents a different question from the comparative effectiveness of transport strategies. Therefore, we aimed to describe the time-dependent probability of subsequent on-scene ROSC among adults with cardiogenic OHCA and an initial shockable rhythm under existing Japanese EMS practice. We also examined favorable neurological outcomes according to the timing of on-scene ROSC and descriptively evaluated these patterns according to sex, age, witnessed status, and bystander CPR.

## 2. Materials and Methods

### 2.1. Study Design

This nationwide retrospective descriptive cohort study used a database comprising two datasets: Japan’s national Utstein database and all emergency transport records.

### 2.2. EMS System in Japan

Japan’s emergency medical system, organized by local governments, uses a nationwide unified 119 emergency call system that dispatches the nearest available ambulance from the local fire departments. Ambulances carry an EMS crew of three, at least one of whom is a nationally certified emergency life-saving technician (ELT). An ELT can perform intravenous access, administer epinephrine intravenously, and establish an advanced airway under the direction of an online physician. All resuscitation procedures by EMS are regulated by local medical control protocols that are based on national resuscitation protocols issued by the Fire and Disaster Management Agency of the Ministry of Internal Affairs and Communications of Japan.

### 2.3. Data Collection

Data from Japan’s Utstein database and emergency transport records between January 2017 and December 2022 were collected from all fire departments in Japan. The Utstein dataset was recorded along with the Utstein guidelines. Both datasets include patients’ age, sex, area codes, and time of the emergency call; however, the Utstein dataset does not include on-scene time. To evaluate on-scene time for OHCA, the datasets were merged based on emergency call acceptance date/time, age, sex, and area codes. Duplicate data were excluded.

### 2.4. Inclusion and Exclusion Criteria

**Figure 1:**
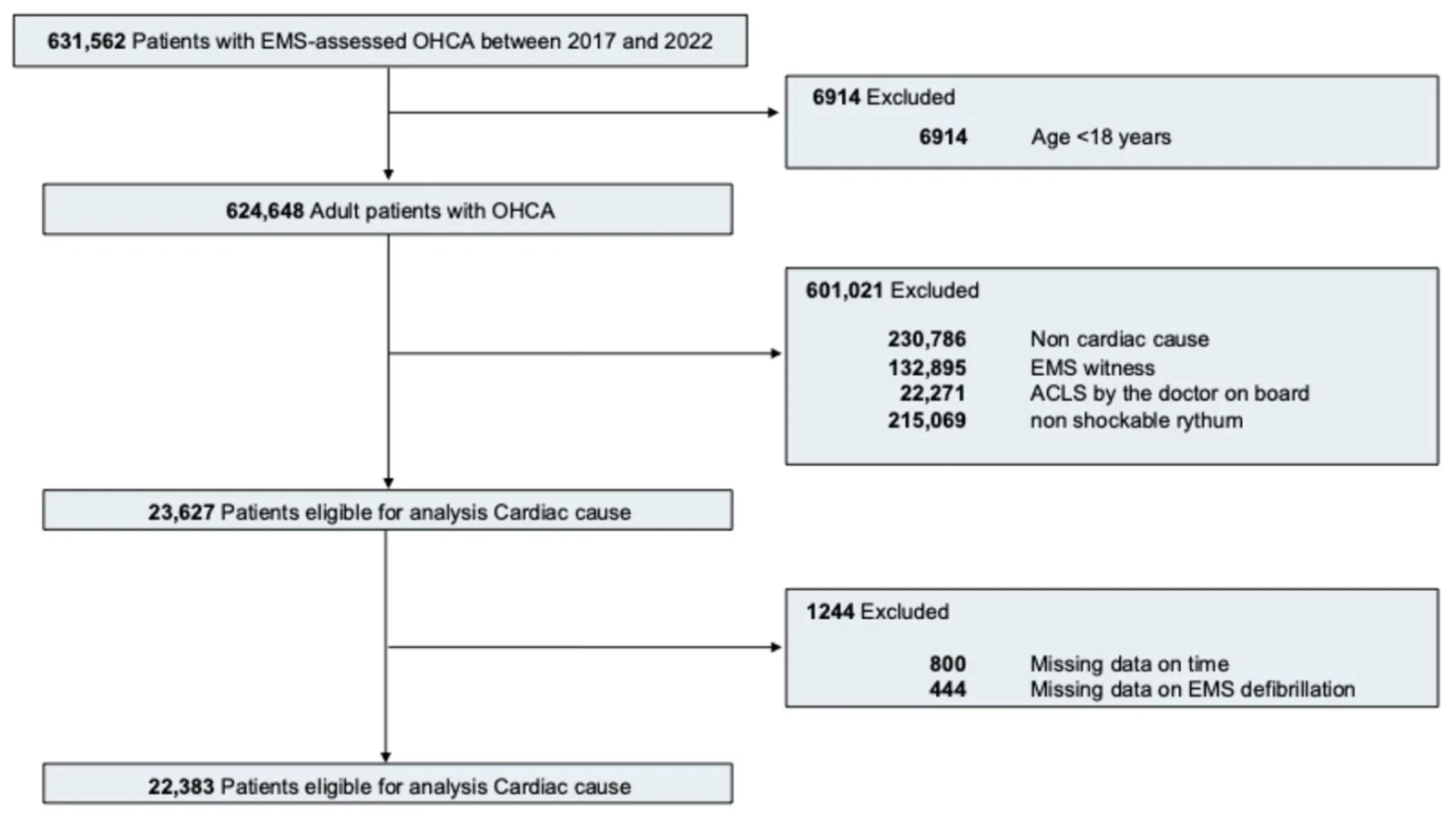
Flowchart of study participants with cardiogenic out-of-hospital cardiac arrest eligible for defibrillation.

Adults aged 18 years or older with OHCA of cardiac cause and an initial shockable rhythm were included. We excluded patients aged <18 years, patients with a non-cardiac cause of cardiac arrest, cases classified as EMS-assessed, cases in which advanced cardiovascular life support (ACLS) was performed by a doctor on board, and cases with a non-shockable initial rhythm, in accordance with the study flowchart (Figure 1). We then excluded cases with missing time data or missing EMS defibrillation data.

### 2.5. Outcomes

The primary outcome was on-scene ROSC. The secondary outcome was a favorable neurological outcome at one month, defined as CPC 1 or 2. One-month survival was also described as a clinical outcome.

### 2.6. Statistical Analysis

Data are presented as medians and interquartile ranges (IQRs) for continuous variables and numbers and percentages for categorical variables. Patients who achieved on-scene ROSC were compared with those who did not achieve on-scene ROSC using the Mann–Whitney U test for continuous variables and the chi-square test for categorical variables, with a significance threshold of *p* < 0.05. EMS contact with the patient was defined as time zero. For each time point t after EMS contact, the observed probability of subsequent on-scene ROSC was calculated using as the numerator the number of patients who achieved on-scene ROSC at or after time t and as the denominator the number of patients who had not achieved on-scene ROSC before time t. This measure therefore represents the observed probability of subsequent on-scene ROSC among patients without prior on-scene ROSC and does not represent the probability of ROSC occurring specifically during that one-minute interval. Among patients who achieved on-scene ROSC, neurological outcome was evaluated according to ROSC timing. For each time point t, the denominator was the number of patients who achieved on-scene ROSC at or after time t, and the numerator was the number of those patients with CPC 1 or 2 at one month. These analyses were descriptively stratified by sex, age (<75 vs. ≥75 years), witnessed status, and bystander CPR. Age was dichotomized at 75 years based on previous reports examining age-specific resuscitation duration and prognosis in OHCA [10,11]. The age categorization was used for descriptive subgroup analysis and was not intended to define a treatment threshold. Wilson 95% confidence intervals were calculated for observed proportions. Observed estimates were connected by lines for visualization only; no model-based smoothing was applied. Because the timing of initial defibrillation could influence both ROSC timing and neurological outcome, a sensitivity analysis using the same approach as the subgroup analysis described above was performed among patients who achieved on-scene ROSC and received their first EMS defibrillation within 1 min after EMS contact. To assess potential linkage bias, baseline characteristics were compared between the original Utstein dataset and the merged dataset; effect sizes were summarized using area-under-the-curve values for continuous variables and Cramer’s V for categorical variables (Table S1). All statistical analyses were performed using Python version 3.11 (Python Software Foundation, Wilmington, DE, USA).

## 3. Results

Among the 778,807 OHCAs during the study period, 631,562 cases (81.1%) were successfully merged. Although several comparisons between the original Utstein and merged datasets reached statistical significance because of the large sample size, the effect sizes were negligible, with area-under-the-curve values close to 0.5 for continuous variables and Cramer’s V values < 0.01 for categorical variables (Table S1). Of the 631,562 merged cases, 22,383 met the eligibility criteria for the present analysis (Figure 1).

The characteristics of the study subjects are shown in Table 1. Among these, 5095 cases achieved ROSC at the scene (group with on-scene ROSC) and 17,288 did not (group without on-scene ROSC). The group with on-scene ROSC, compared to the group without on-scene ROSC, was younger (66 years vs 69 years, *p* < 0.001), had cases that were more likely to be witnessed (4483 [88.0%] vs. 13,655 [79.0%], *p* < 0.001), and had cases where patients were more likely to receive bystander chest compressions (3552 [69.7%] vs. 10,246 [59.3%], *p* < 0.001). On the other hand, prehospital airway device placement and epinephrine administration were frequent in the group without on-scene ROSC (9196 [53.2%] vs. 1461 [28.7%], *p* < 0.001, and 7885 [45.6%] vs. 925 [18.2%], *p* < 0.001, respectively). The median prehospital time intervals (response time, on-scene time, and transport time) were comparable between the two groups (group with on-scene ROSC vs. group without on-scene ROSC: 7 min vs. 8 min, 13 min vs. 12 min, and 9 min vs. 9 min, respectively). A favorable neurological outcome, defined as CPC 1/2, was achieved in 4983 cases, and was more frequent in the group with on-scene ROSC (3182 [62.4%] vs. 1801 [10.4%], *p* < 0.001).

**Figure 2:**
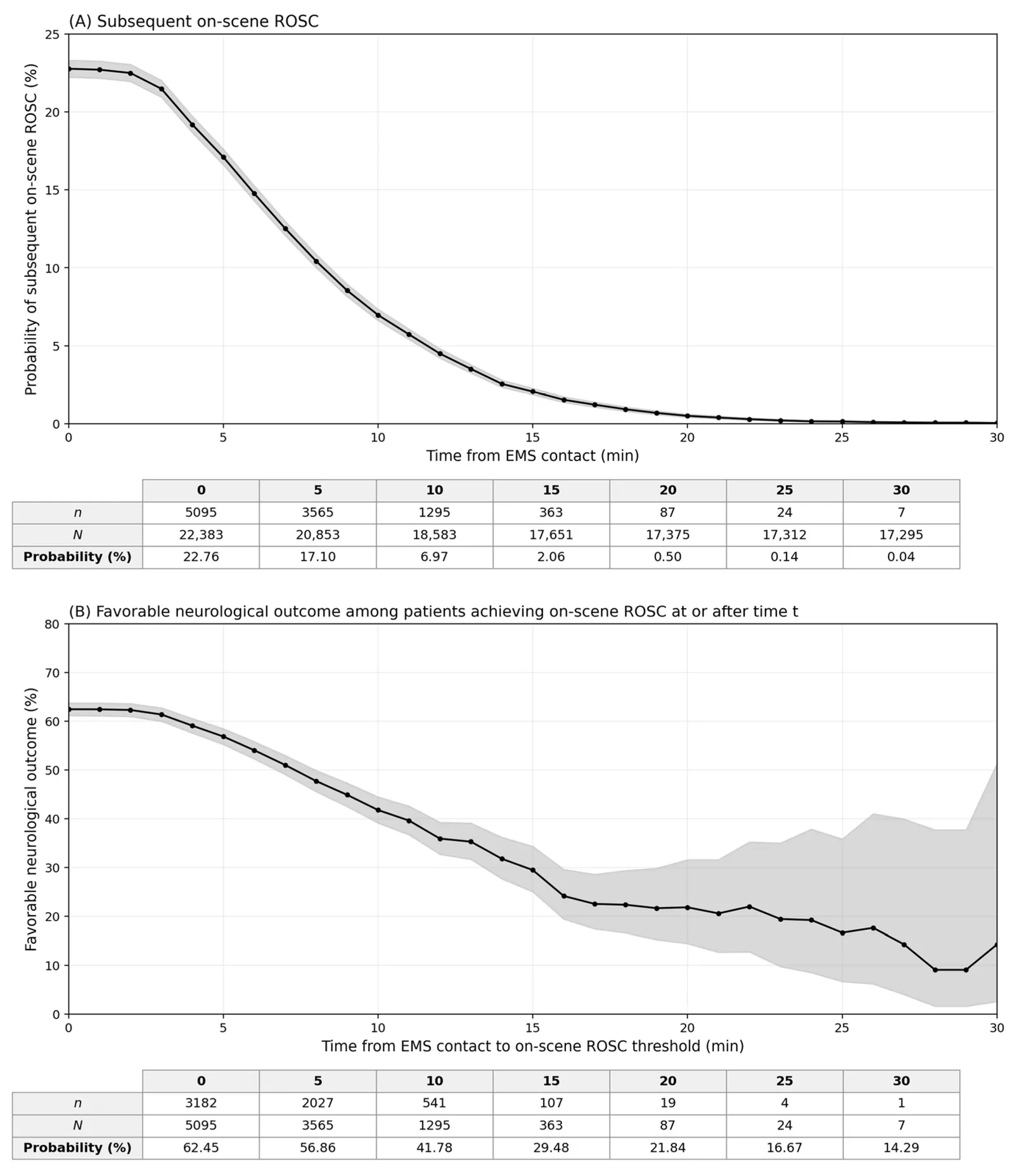
Time-dependent probabilities of subsequent on-scene ROSC and favorable neurological outcome according to time after EMS contact. (**A**) For each time point after EMS contact, the numerator was the number of patients who achieved on-scene ROSC at or after that time point, and the denominator was the number of patients who had not achieved on-scene ROSC before that time point. (**B**) Among patients who achieved on-scene ROSC, the numerator was the number with a favorable neurological outcome (CPC 1 or 2 at one month) who achieved on-scene ROSC at or after each time point, and the denominator was the total number of patients who achieved on-scene ROSC at or after that time point. Points represent observed probabilities and shaded areas indicate 95% confidence intervals. Lines connecting adjacent estimates are shown for visual presentation only and do not represent model-based smoothing. ROSC, return of spontaneous circulation; EMS, emergency medical services; CPC, Cerebral Performance Category.

Figure 2A shows the time-dependent probability of subsequent on-scene ROSC among the 22,383 eligible patients. The observed probability decreased markedly with elapsed time after EMS contact. At 10 min, 1295 of 18,583 patients without prior on-scene ROSC subsequently achieved on-scene ROSC (6.97%; 95% CI, 6.61–7.34%). At 20 min, the corresponding probability was 0.50% (87/17,375; 95% CI, 0.41–0.62%). Figure 2B shows the overall probability of a favorable neurological outcome among patients who achieved on-scene ROSC at or after each time point. This probability was 62.45% (3182/5095; 95% CI, 61.11–63.77%) at 0 min, 41.78% (541/1295; 95% CI, 39.12–44.48%) at 10 min, and 21.84% (19/87; 95% CI, 14.45–31.61%) at 20 min. Numerators and denominators at 5-min intervals are shown in Figure 2.

**Figure 3:**
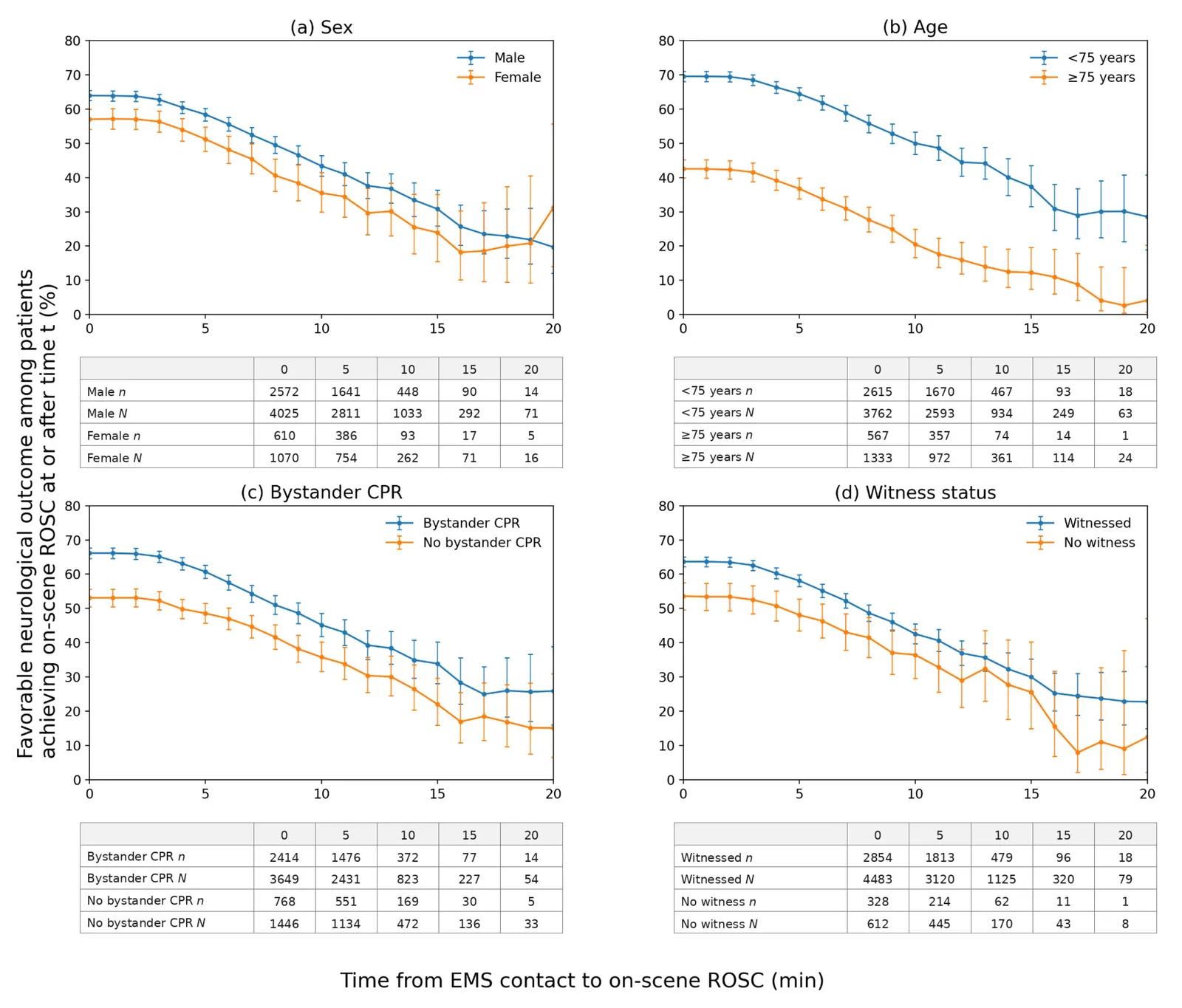
Probability of a favorable neurological outcome according to the timing of on-scene ROSC, stratified by patient characteristics. The figure shows the probability of a favorable neurological outcome among patients who achieved on-scene ROSC at or after each time point, stratified by (**a**) sex, (**b**) age, (**c**) bystander CPR, and (**d**) witnessed status. For each time point, the numerator was the number of patients with a favorable neurological outcome (CPC 1 or 2 at one month) who achieved on-scene ROSC at or after that time point, and the denominator was the total number of patients who achieved on-scene ROSC at or after that time point within the corresponding subgroup. Points represent observed estimates, and error bars indicate 95% confidence intervals. Lines connecting adjacent estimates are shown for visual presentation only and do not represent model-based smoothing. ROSC, return of spontaneous circulation; CPR, cardiopulmonary resuscitation.

Figure 3 shows the probability of a favorable neurological outcome according to the timing of on-scene ROSC among the 5095 patients who achieved on-scene ROSC, stratified by sex, age, bystander CPR, and witnessed status. Across all subgroups, the observed probability of a favorable neurological outcome generally decreased with later ROSC timing. The separation between subgroup estimates was most pronounced for age. At 10 min after EMS contact, favorable neurological outcomes were observed in 50.0% of patients aged <75 years who achieved on-scene ROSC at or after that time point (467/934) and in 20.5% of those aged ≥75 years (74/361). Estimates at later time points were based on progressively smaller denominators and therefore had wider 95% confidence intervals.

**Figure 4:**
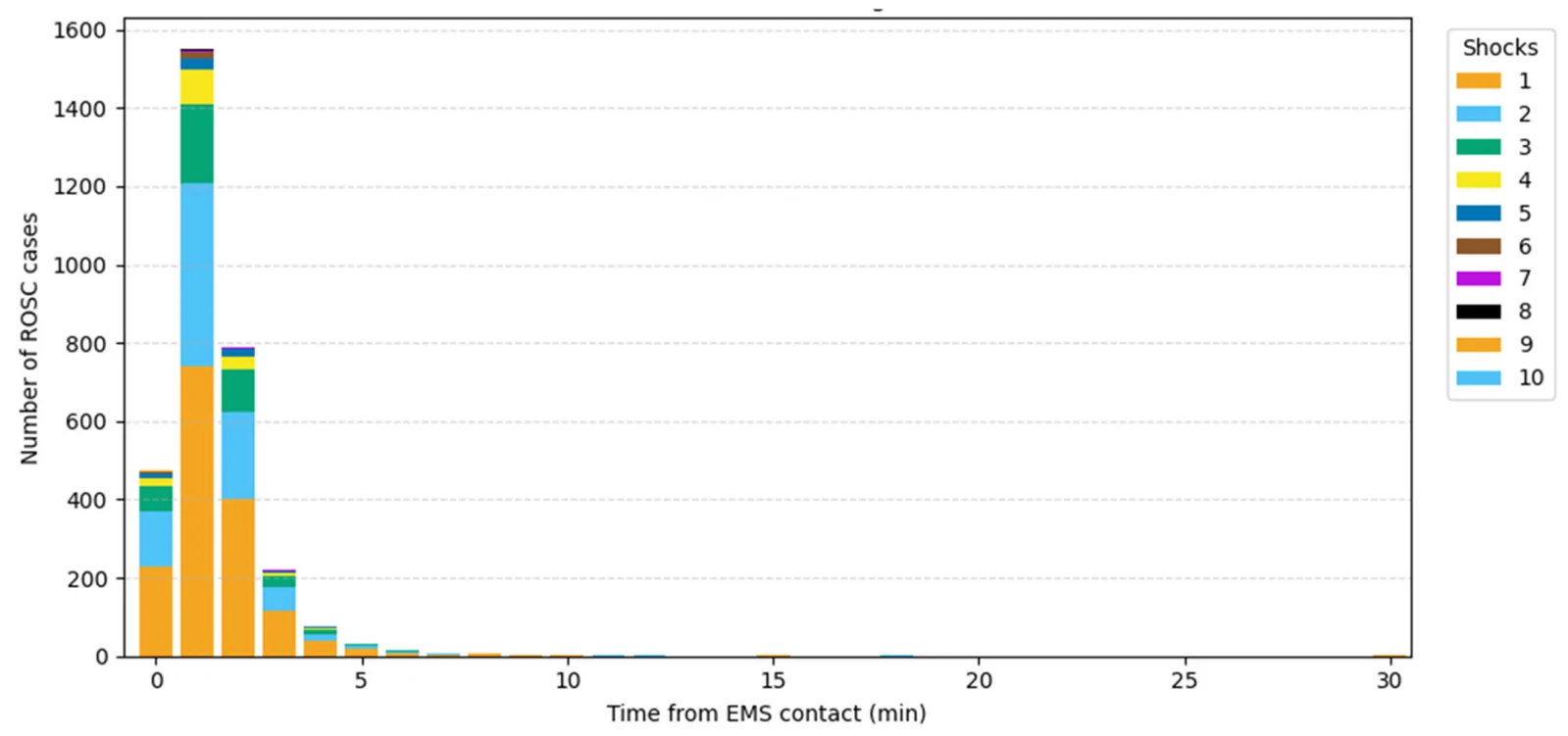
Distribution of time to first EMS defibrillation and the number of defibrillation attempts among patients achieving on-scene ROSC with favorable neurological outcomes. The figure shows the distribution of time to first EMS defibrillation and the number of defibrillation attempts among 3182 patients who achieved on-scene ROSC with a favorable neurological outcome. The distribution peaked at 1 min after EMS contact, and 2026 patients (63.6%) received their first EMS defibrillation within 1 min. ROSC, return of spontaneous circulation; EMS, emergency medical services.

Since the timing of initial defibrillation may influence both ROSC timing and neurological outcome, we performed a sensitivity analysis restricted to patients who achieved on-scene ROSC and received their first EMS defibrillation within 1 min after EMS contact. Figure 4 shows the distribution of time to first EMS defibrillation among the 3182 patients who achieved on-scene ROSC with a favorable neurological outcome. Of these patients, 2026 (63.6%) received their first EMS defibrillation within 1 min after EMS contact. Overall, 3128 patients who achieved on-scene ROSC received their first EMS defibrillation within 1 min after EMS contact, of whom 2026 had a favorable neurological outcome. When the analysis shown in Figure 3 was repeated in this restricted cohort, including stratification by age, sex, witness status, and bystander CPR, the overall temporal patterns were broadly consistent with those observed in the primary analysis (Figure S1).

## 4. Discussion

This study demonstrates that, in OHCA with an initial shockable rhythm, the observed probability of subsequent on-scene ROSC under current resuscitation practices in Japan decreased markedly with elapsed time after EMS contact. At 10 min, the probability was 6.97%, and at 20 min it was 0.50%. Among patients who achieved on-scene ROSC, the probability of a favorable neurological outcome also decreased with later ROSC timing. These findings characterize temporal patterns observed under existing Japanese EMS practice and should not be interpreted as defining a causal effect of remaining on scene.

Previous studies have examined the association between resuscitation duration and outcome and have reported decreasing probabilities of favorable outcomes with prolonged resuscitation [4,5,6]. Our findings are consistent with the general observation that both ROSC and favorable neurological outcome become less frequent with increasing resuscitation time. However, the present analysis estimates the observed probability of subsequent on-scene ROSC among patients without prior on-scene ROSC and does not establish a fixed upper limit for on-scene resuscitation.

Several studies have directly compared intra-arrest transport with continued on-scene resuscitation [7,8,9]. Grunau et al. reported no advantage of intra-arrest transport in a large propensity-matched cohort [7], whereas other studies have suggested that the association may vary according to rhythm or scene duration [8,9]. Importantly, the present study did not compare transport strategies. Therefore, our findings should not be interpreted as evidence favoring either prolonged on-scene resuscitation or earlier intra-arrest transport.

In the descriptive subgroup analysis, patients aged <75 years showed higher observed probabilities of favorable neurological outcomes at later ROSC times than patients aged ≥75 years. For example, at 10 min after EMS contact, the probability of a favorable neurological outcome among patients achieving on-scene ROSC at or after that time point was 50.0% in patients aged <75 years and 20.5% in those aged ≥75 years. This finding suggests that younger age may be associated with a greater likelihood of favorable neurological recovery even when on-scene ROSC is achieved later. This observation is consistent with previous findings by Funada et al., who reported that the duration of prehospital CPR compatible with favorable neurological outcomes tended to be longer in younger patients who achieved prehospital ROSC [10]. However, these findings should be interpreted cautiously. The age cutoff of 75 years was used for descriptive stratification in accordance with previous reports, including a study by Funada et al. that identified patients aged <75 years with an initial shockable rhythm as a prognostically distinct subgroup among patients without prehospital ROSC [11], and was not intended to represent a biological threshold or a criterion for treatment selection. Moreover, the present study was not designed to determine whether younger patients should undergo prolonged on-scene resuscitation or whether transport should be delayed. Therefore, the observed age-related differences should not be interpreted as evidence supporting an age-based strategy for the duration of on-scene resuscitation or the timing of intra-arrest transport. The subgroup curves according to witnessed status and bystander CPR showed broadly similar declining temporal patterns; however, visual similarity alone does not establish the absence of clinically relevant associations.

Initial defibrillation timing is an important determinant of outcome in shockable OHCA. Among the 3182 patients with on-scene ROSC and a favorable neurological outcome, 2026 (63.6%) received their first EMS defibrillation within 1 min after EMS contact (Figure 4). In the sensitivity analysis restricted to all 3128 patients who achieved on-scene ROSC and received first EMS defibrillation within 1 min, the temporal patterns were broadly consistent with those observed in the primary analysis (Figure S1). This finding suggests that the descriptive temporal patterns were not solely explained by delayed initial EMS defibrillation. Another factor that may influence these temporal patterns is the quality of prehospital CPR, which could not be evaluated in our cohort. Several animal experiments and human observational studies indicate that peri-defibrillation CPR quality, including a high chest compression fraction and minimized peri-shock pause, can increase the chance of defibrillation success [12,13,14,15,16].

### Limitations

This study has several limitations. First, because this was a retrospective observational study, causal inference was not possible and unmeasured confounding may remain. Second, the Figure 2 denominator included patients who had not achieved on-scene ROSC before each time point; some of these patients may already have departed the scene without ROSC. Scene departure without ROSC is an informative event rather than random censoring, and patients remaining eligible at later time points therefore represent a selected population shaped by patient characteristics, response to resuscitation, EMS decisions, local protocols, and access to hospital-based treatment. Accordingly, Figure 2 does not estimate what would have occurred had all patients remained on scene. Third, this study did not compare the causal effects of continued on-scene resuscitation and intra-arrest transport and therefore cannot determine the preferred transport strategy. Fourth, the neurological outcome analyses in Figure 3 and Figure S1 were conditional on achieving on-scene ROSC and cannot be generalized to all patients who remained in cardiac arrest at the corresponding time point. One-month neurological outcome may also have been influenced by subsequent transport and in-hospital treatment. Fifth, the number of patients contributing to later time points decreased substantially, resulting in wider confidence intervals and less stable subgroup estimates. Sixth, although 81.1% of the original registry could be linked, effect-size comparisons between the original and merged datasets suggested only negligible differences (Table S1); nevertheless, residual linkage bias cannot be excluded. Seventh, 1244 cases were excluded because of missing time variables or EMS defibrillation data, which may have introduced selection bias. Finally, regional differences in EMS protocols and scene environments, including high-rise buildings, may have influenced on-scene duration and resuscitation processes.

## 5. Conclusions

Among patients with OHCA and an initial shockable rhythm, the observed probability of subsequent on-scene ROSC decreased markedly with elapsed time after EMS contact under existing Japanese EMS practice. Among patients who achieved on-scene ROSC, the probability of a favorable neurological outcome also decreased with later ROSC timing. Younger patients showed more favorable neurological outcomes at later ROSC times than older patients; however, this finding does not establish an age-based criterion for prolonged on-scene resuscitation or transport decisions.

## Figures and Tables

**Table 1:** Characteristics of the study participants.

	Total	On-Scene ROSC	Non-ROSC	*p*-Values
	(*n* = 22,383)	(*n* = 5095)	(*n* = 17,288)	
Male sex, *n* (%)	17,881 (79.9)	4025 (79.0)	13,856 (80.1)	0.07
Age, years (IQR)	69 (57–78)	66 (55–75)	69 (58–79)	<0.001
Witness, *n* (%)	18,138 (81.0)	4483 (88.0)	13,655 (79.0)	<0.001
Bystander chest compressions, *n* (%)	13,798 (61.6)	3552 (69.7)	10,246 (59.3)	<0.001
				
Prehospital use of				
advanced airway devices, *n* (%)	10,657 (47.6)	1461 (28.7)	9196 (53.2)	<0.001
Epinephrine administration, *n* (%)	8810 (39.4)	925 (18.2)	7885 (45.6)	<0.001
				
Response time, min (IQR)	8 (6–10)	7 (6–9)	8 (6–10)	<0.001
On-scene time, min (IQR)	12 (9–16)	13 (10–17)	12 (9–16)	<0.001
Transport time, min (IQR)	9 (6–14)	9 (6–14)	9 (6–14)	<0.001
				
Survival at 1 month, *n* (%)	7509 (33.5)	3987 (78.3)	3522 (20.4)	<0.001
Favorable neurological outcome, *n* (%)	4983 (22.2)	3182 (62.4)	1801 (10.4)	<0.001

IQR, interquartile range; ROSC, return of spontaneous circulation.

## Data Availability

The datasets used and/or analyzed during the current study are available from the corresponding author on reasonable request.

## Supplementary Materials

The following supporting information can be downloaded at https://www.mdpi.com/article/10.3390/jcm15218484/s1, Figure S1: Sensitivity analysis of the probability of a favorable neurological outcome according to the timing of on-scene ROSC among patients receiving first EMS defibrillation within 1 min after EMS contact; Table S1: Characteristics of the study subjects: original versus combined datasets.

## Abbreviations

OHCA	out-of-hospital cardiac arrest
EMS	emergency medical services
ELT	emergency life-saving technician
ROSC	return of spontaneous circulation
CPC	cerebral performance category

## References

[1] Igeta R., Otaka S., Imaizumi T., Kajino K., Khoujah D., Ito F., Endo T., Chiba T., Ikeda S., Shiga T. (2024). Regional comparison in cerebral performance outcome of out-of-hospital cardiac arrest: The All-Japan Utstein Registry. Cureus.

[2] Martin S.S., Aday A.W., Allen N.B., Almarzooq Z.I., Anderson C.A.M., Arora P., Avery C.L., Baker-Smith C.M., Bansal N., Beaton A.Z. (2025). 2025 Heart disease and stroke statistics: A report of US and global data from the American Heart Association. Circulation.

[3] Matsuyama T., Kiyohara K., Kitamura T., Nishiyama C., Kiguchi T., Iwami T. (2022). Public-access defibrillation and favorable neurological outcome after out-of-hospital cardiac arrest during the COVID-19 pandemic in Japan. Crit. Care.

[4] Matsuyama T., Ohta B., Kiyohara K., Kitamura T. (2022). Cardiopulmonary resuscitation duration and favorable neurological outcome after out-of-hospital cardiac arrest: A nationwide multicenter observational study in Japan (the JAAM-OHCA registry). Crit. Care.

[5] Reynolds J.C., Frisch A., Rittenberger J.C., Callaway C.W. (2013). Duration of resuscitation efforts and functional outcome after out-of-hospital cardiac arrest: When should we change to novel therapies?. Circulation.

[6] Coute R.A., Nathanson B.H., Kurz M.C., McNally B., Mader T.J., CARES Surveillance Group (2021). The association between scene time interval and neurologic outcome following adult bystander witnessed out-of-hospital cardiac arrest. Am. J. Emerg. Med..

[7] Grunau B., Kime N., Leroux B., Rea T., Van Belle G., Menegazzi J.J., Kudenchuk P.J., Vaillancourt C., Morrison L.J., Elmer J. (2020). Association of intra-arrest transport vs. continued on-scene resuscitation with survival to hospital discharge among patients with out-of-hospital cardiac arrest. JAMA.

[8] Shimomura Y., Komukai S., Zha L., Kiyohara K., Shiozumi T., Matsuyama T., Kitamura T. (2026). Intra-arrest transport versus continued on-scene resuscitation in patients with out-of-hospital cardiac arrest by first documented rhythm: A nationwide cohort study using time-dependent propensity score sequential matching. Crit. Care.

[9] Burns B., Marschner I.C., Buscher H., Coggins A., Oliver M., Maruno K., McNulty R., Hawkins S., Facer R., Pradhananga B. (2025). Expedited transfer from the scene for refractory out-of-hospital cardiac arrest in Australia: A prospective, multicentre, parallel, open label, randomised clinical trial. Lancet Respir. Med..

[10] Funada A., Goto Y., Tada H., Teramoto R., Shimojima M., Hayashi K., Yamagishi M. (2017). Age-specific differences in the duration of prehospital cardiopulmonary resuscitation administered by emergency medical service providers necessary to achieve favorable neurological outcome after out-of-hospital cardiac arrest. Circ. J..

[11] Funada A., Goto Y., Tada H., Teramoto R., Shimojima M., Hayashi K., Kawashiri M.-A., Yamagishi M. (2018). Duration of cardiopulmonary resuscitation in patients without prehospital return of spontaneous circulation after out-of-hospital cardiac arrest: Results from a severity stratification analysis. Resuscitation.

[12] Yu T., Weil M.H., Tang W., Sun S., Klouche K., Povoas H., Bisera J. (2002). Adverse outcomes of interrupted precordial compression during automated defibrillation. Circulation.

[13] Steen S., Liao Q., Pierre L., Paskevicius A., Sjöberg T. (2003). The critical importance of minimal delay between chest compressions and subsequent defibrillation: A haemodynamic explanation. Resuscitation.

[14] Olsen J.A., Brunborg C., Steinberg M., Persse D., Sterz F., Lozano M., Westfall M., Travis D.T., Lerner E.B., Brouwer M.A. (2015). Pre-shock chest compression pause effects on termination of ventricular fibrillation/tachycardia and return of organized rhythm within mechanical and manual cardiopulmonary resuscitation. Resuscitation.

[15] Iversen B.N., Meilandt C., Væggemose U., Terkelsen C.J., Kirkegaard H., Fjølner J. (2021). Pre-charging the defibrillator before rhythm analysis reduces hands-off time in patients with out-of-hospital cardiac arrest with shockable rhythm. Resuscitation.

[16] Christenson J., Andrusiek D., Everson-Stewart S., Kudenchuk P., Hostler D., Powell J., Callaway C.W., Bishop D., Vaillancourt C., Davis D. (2009). Chest compression fraction determines survival in patients with out-of-hospital ventricular fibrillation. Circulation.

