# Supplementary material for: On-Scene Time-Dependent Probabilities of Return of Spontaneous Circulation and Favorable Neurological Outcome in Cases of Out-of-Hospital Cardiac Arrest with an Initial Shockable Rhythm: A Retrospective Study Using the National Utstein Data in Japan
Source: J Clin Med. 2026 Oct 31;15(21):8484. doi: 10.3390/jcm15218484 (PMC13763627; doi:10.3390/jcm15218484)
Supplement: Supplementary file 1 [file jcm-15-08484-s001.zip › FigureS1.pdf]

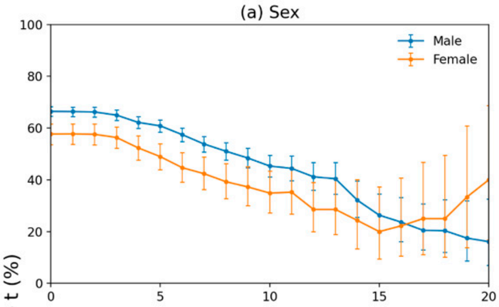

	0	5	10	15	20
Male *n*	1681	993	247	34	5
Male *N*	2530	1632	545	129	31
Female *n*	345	190	45	6	4
Female *N*	598	388	129	30	10

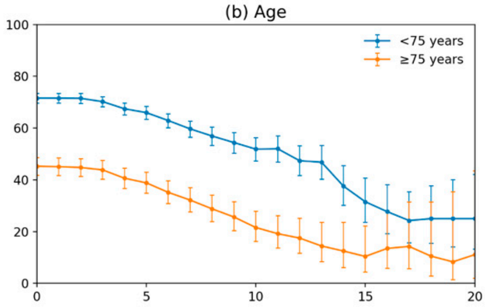

	0	5	10	15	20
<75 years *n*	1660	969	251	35	8
<75 years *N*	2319	1469	484	111	32
≥75 years *n*	366	214	41	5	1
≥75 years *N*	809	551	190	48	9

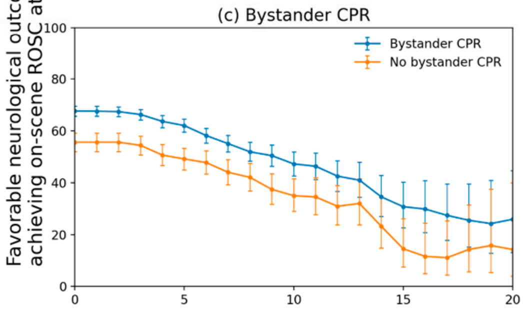

	0	5	10	15	20
Bystander CPR *n*	1607	914	216	32	7
Bystander CPR *N*	2375	1473	457	104	27
No bystander CPR *n*	419	269	76	8	2
No bystander CPR *N*	753	547	217	55	14

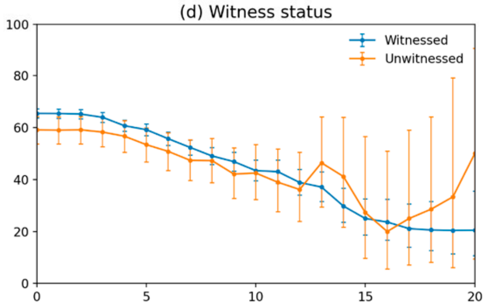

	0	5	10	15	20
Witnessed *n*	1825	1066	258	37	8
Witnessed *N*	2788	1801	594	148	39
Unwitnessed *n*	201	117	34	3	1
Unwitnessed *N*	340	219	80	11	2

Time from EMS contact to on-scene ROSC (min)

Figure S1. Sensitivity analysis of the probability of a favorable neurological outcome according to the timing of on-scene ROSC among patients receiving first EMS defibrillation within 1 min after EMS contact.

The analysis included patients who achieved on-scene ROSC and received their first EMS defibrillation within 1 min after EMS contact. For each time point, the numerator was the number of patients with a favorable neurological outcome (CPC 1 or 2 at one month) who achieved on-scene ROSC at or after that time point, and the denominator was the total number of patients who achieved on-scene ROSC at or after that time point within each subgroup. Estimates are shown according to (a) sex, (b) age, (c) bystander CPR, and (d) witnessed status. Error bars indicate 95% confidence intervals. Lines connecting adjacent estimates are shown for visual presentation only and do not represent model-based smoothing.
