# Supplementary material for: On-Scene Time-Dependent Probabilities of Return of Spontaneous Circulation and Favorable Neurological Outcome in Cases of Out-of-Hospital Cardiac Arrest with an Initial Shockable Rhythm: A Retrospective Study Using the National Utstein Data in Japan
Source: J Clin Med. 2026 Oct 31;15(21):8484. doi: 10.3390/jcm15218484 (PMC13763627; doi:10.3390/jcm15218484)
Supplement: Supplementary file 1 [file jcm-15-08484-s001.zip › Table S1.pdf]

Table S1. Characteristics of the study subjects: original versus combined datasets.

	Original data (*n*=778,807)	Combined data (*n*=631,562)	p values	effect size
Male, *n*(%)	447,236 (57.4)	362,380 (57.4)	0.572*	<0.001§
Age, yrs(IQR)	80(69-87)	80(69-87)	p<0.01**	0.502¶
Witness, *n*(%)	320,854 (41.2)	258,691(41.0)	p<0.01*	<0.01§
Bystander chest compression, *n*(%)	390,720 (50.2)	325,439 (51.5)	p<0.01*	<0.01§
Initial shockable rhythm, *n*(%)	46481 (6.0)	37610 (6.0)	0.742*	<0.001§
Prehospital				
advanced airway devices, *n*(%)	324,686(41.7)	274,949 (43.5)	p<0.01*	<0.01§
epinephrine administration, *n*(%)	200,445 (25.7)	169,422 (26.8)	p<0.01*	<0.01§
Response time, min(IQR)	8 (6-10)	8 (6-10)	p<0.01**	0.532¶
Transportation time, min(IQR)	33 (27-41)	33 (27-41)	p<0.01**	0.492¶
Survival at 1 month, *n*(%)	48,275 (6.2)	40,533(6.4)	0.246*	<0.01§
Favorable neurological outcome, *n*(%)	27,756 (3.7)	23,129 (3.7)	p<0.01*	<0.01§

IQR; interquartile range, *Chi-square test, **Mann-Whitney U test, §Cramer's V, ¶Area Under the Curve.

Because the sample sizes of the two datasets (Original and combined data) were very large , even slight differences yielded extremely small p-values, resulting in statistical significance. Therefore, in addition to evaluating statistical significance, we assessed the magnitude of the differences by calculating the area under curve (AUC) for continuous variables and Cramer's V for categorical variables. The AUC values were approximately 0.5, and the Cramer's V values were all less than 0.01, indicating extremely small effect sizes. Although statistically significant differences were observed, these effect sizes

suggested that the differences were negligible in practical and clinical terms. Therefore, we further analyzed the combined dataset.
